# Supplementary material for: A Global Screen for Assembly State Changes of the Mitotic Proteome by SEC-SWATH-MS
Source: Cell Syst. 2020 Feb 26;10(2):133–155.e6. doi: 10.1016/j.cels.2020.01.001 (PMC7042714; doi:10.1016/j.cels.2020.01.001)

Q8WZA9 | IRGQ_HUMAN | IRGQ IRGQ1 FKSG27
Monomer MW [kDa]: 62.717 Monomer expected elution fraction: 44

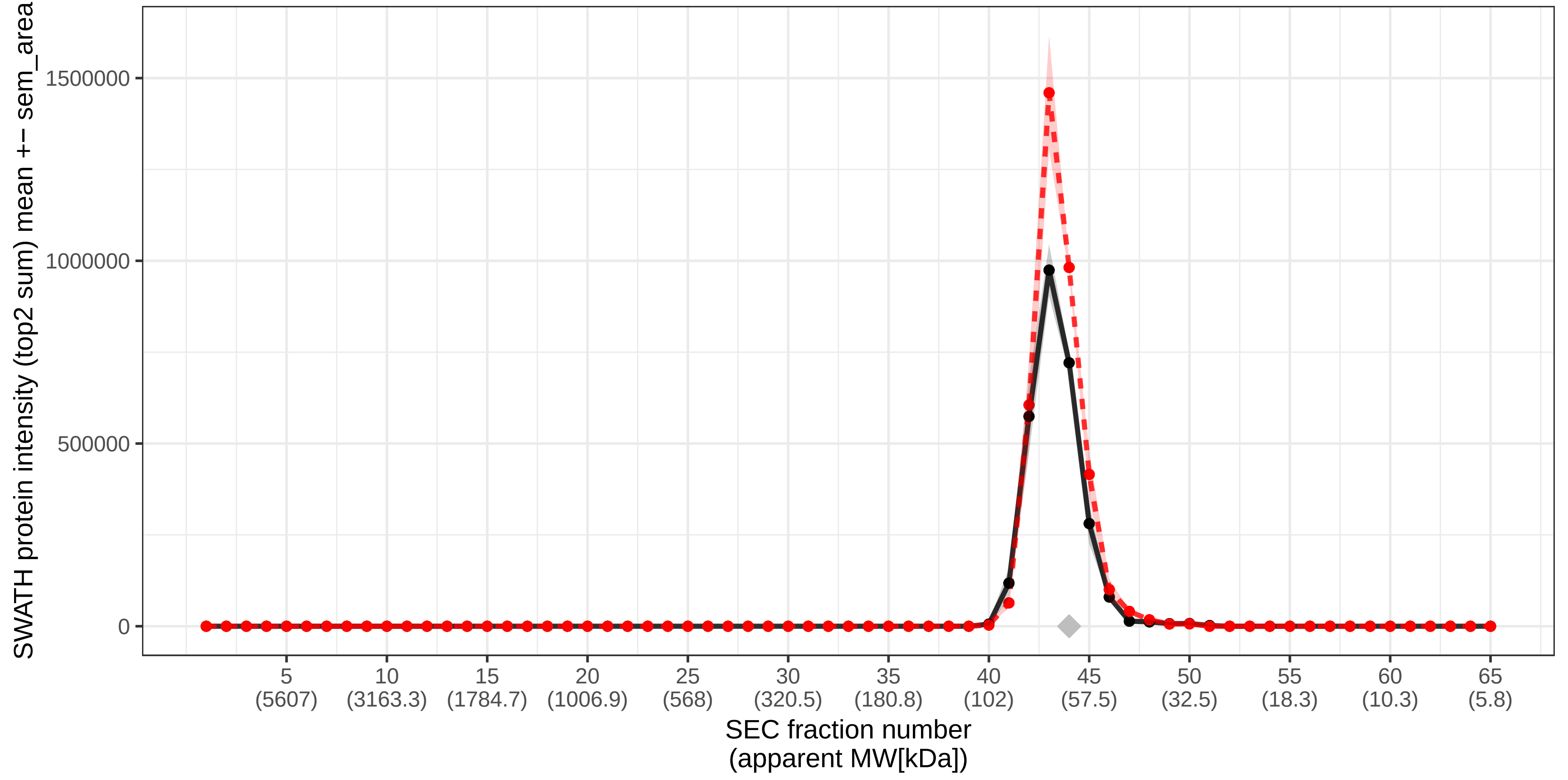

Supplement: Data S1. SEC-SWATH-MS Protein Chromatograms, Related to Figure 1 [file mmc6.zip › SECchrom_Q8WZA9_IRGQ_HUMAN_IRGQ_IRGQ1_FKSG27.pdf]
